# Unusual Emission of Polystyrene-Based Alternating Copolymers Incorporating Aminobutyl Maleimide Fluorophore-Containing Polyhedral Oligomeric Silsesquioxane Nanoparticles

**DOI:** 10.3390/polym9030103

**Published:** 2017-03-15

**Authors:** Mohamed Gamal Mohamed, Yu-Ru Jheng, Shu-Ling Yeh, Tao Chen, Shiao-Wei Kuo

**Affiliations:** 1 Department of Materials and Optoelectronic Science, National Sun Yat-Sen University, Kaohsiung 80424, Taiwan; mgamal.eldin12@yahoo.com (M.G.M.); m043100015@student.nsysu.edu.tw (Y.-R.J.); 2 Department of Applied Chemistry, National Chiao Tung University, Hsinchu 300, Taiwan; ShuLingYeh@itri.org.tw; 3 Ningbo Institute of Material Technology and Engineering, Key Laboratory of Graphene Technologies and Applications of Zhejiang Province, Chinese Academy of Science, Zhongguan West Road 1219, Ningbo 315201, China; tao.chen@nimte.ac.cn; 4 Department of Medicinal and Applied Chemistry, Kaohsiung Medical University, Kaohsiung 807, Taiwan

**Keywords:** unusual emission, POSS, hydrogen bonding, alternative copolymer

## Abstract

In this study, we synthesized an unusual 2-aminobutyl maleimide isobutyl polyhedral oligomeric silsesquioxane (MIPOSS-NHBu) monomer lacking conventional fluorescent groups. We then prepared poly(styrene-*alt*-2-aminobutyl maleimide isobutyl POSS) [poly(S-*alt*-MIPOSS-NHBu)] and poly(4-acetoxystyrene-*alt*-2-aminobutyl maleimide isobutyl POSS) [poly(AS-*alt*-MIPOSS-NHBu)] copolymers through facile free radical copolymerizations using azobisisobutyronitrile as the initiator and tetrahydrofuran as the solvent. A poly(4-hydroxystyrene-*alt*-2-aminobutyl maleimide isobutyl POSS) [poly(HS-*alt*-MIPOSS-NHBu)] copolymer was prepared through acetoxyl hydrazinolysis of poly(AS-*alt*-MIPOSS-NHBu). We employed ^1^H, ^13^C, and ^29^Si nuclear magnetic resonance spectroscopy; Fourier transform infrared spectroscopy; differential scanning calorimetry; and photoluminescence spectroscopy to investigate the structures and the thermal and optical properties of the monomers and novel POSS-containing alternating copolymers. Intramolecular hydrogen bonding between the amino and dihydrofuran-2,5-dione group and clustering of the locked C=O groups from the POSS nanoparticles in the MIPOSS-NHBu units restricted the intramolecular motion of the polymer chain, causing it to exhibit strong light emission. As a result, the MIPOSS-NHBu monomer and the poly(AS-*alt*-MIPOSS-NHBu) copolymer both have potential applicability in the detection of metal ions with good selectivity.

## 1. Introduction

In recent years, fluorescent materials and polymers have attracted much attention in academic and industrial fields for their potential applications in optoelectronic devices, organic light emitting diodes, bio-imaging, chemical sensors, biosensors, drug delivery, DNA probing, and protein sensors [1,2,3,4,5,6,7,8,9,10,11]. Most fluorescent organic molecules exhibit a strong emission in dilute solution, but become non-luminescent or weakly emissive in their condensed or aggregated state, a phenomenon known as aggregation-caused quenching [12,13,14,15]. Therefore, synthesized organic luminescent materials and polymers that exhibit strong fluorescence in the solid state have industrial and research applications [16,17,18]. In 2001, Tang and co-workers discovered novel silole derivatives that are highly emissive in the aggregated state and non-emissive in solution. These materials display aggregation-induced emission (AIE) or aggregation-induced enhancement emission (AIEE) [19,20,21,22]. Many reports have suggested ways to enhance the intense fluorescence of organic materials in the solid state, including J-aggregate formation [23], constructing cross-linked polymer networks and dendrimers [24], introducing non-planar structures and bulky substituents [25], restricting intramolecular motions (for AIE mechanism) [26], and inducing excited state intramolecular proton transfer [27,28] and twisted intramolecular charge transfer [29,30].

Several fluorescent polymers lacking benzene or heterocyclic rings have been developed because for their potential for excellent biocompatibility and biodegradability [31,32,33]. For instance, Yang and co-workers noted the unexpected strong emission from monomers and polymers containing 2-thio- and 2-amino-succinimide, caused by the isolation effect [34]. Pucci et al. reported the unexpected fluorescence of polyisobutene-grafted succinic anhydride and imide, ascribed to the aggregation of non-emissive chromophoric C=O groups and intermolecular interactions among the dihydrofuran-2,5-dione groups [35]. Zhao et al. synthesized pure oxygenic nonconjugated poly[(maleic anhydride-*alt*-(vinyl acetate)] through free-radical polymerization; this alternating copolymer exhibited strong light emission and a solvatochromic effect with electron-rich solvents, with the emission behavior originating from the clustering of locked C=O groups [36]. Wu et al. reported the first water-soluble maleimide carrying an NIR-BF_2_-azadipyrromethene (NIR-AZA) as near infrared fluorochrome and this fluorochrome reacted rapidly with biomolecules [37]. Recently, we prepared polystyrene-based alternating copolymers containing maleimide polyhedral oligomeric silsesquioxane (MIPOSS) nanoparticles (NPs) and found that the poly(MIPOSS) homopolymer had greater photoluminescence emission than the POSS-containing alternating copolymers, due to the high crystallinity and the clustering of locked C=O groups of the MIPOSS segments restricting intramolecular motion of the polymer chains [31].

Inorganic/organic hybrid materials and polymers containing POSS units and their derivatives have attracted great attention from research and industrial areas because of the unique properties resulting from the well-defined three-dimensional cubic cages having dimensions of 1–3 nm. The chemical formula of a typical POSS structure is (RSiO_1.5_)_8_; it presents organic functional groups (R) that can be reactive or non-reactive as the outer layer, with an inner core of an inorganic cage structure comprising silicon and oxygen atoms [38,39,40,41,42]. POSS NPs can be classified into three main types: molecular silica and mono- and multifunctional POSS NPs. The incorporation of POSS NPs into a polymeric matrix has been performed through living/controlled polymerization and click chemistry to prepare novel hybrid materials exhibiting good heat-resistance, flammability, and thermal and mechanical properties, and low dielectric constants [43,44,45,46,47,48,49,50]. The self-assembly of POSS-containing hybrid polymers has also been studied. For example, Cheng et al. reported the various morphologies of a giant surfactant (PS-APOSS), from vesicles to wormlike structures to spherical micelles in selective solvents [51]. Zhang et al. investigated the self-assembly of POSS-containing poly(ethylene oxide)s (POSS-PEO) and poly(acrylic acid)s (POSS-PAA) in aqueous solution; they observed that these POSS-containing hybrid polymers formed aggregates having various structures [52]. We have synthesized POSS-containing helical polypeptide copolymers through controlled ring-opening polymerizations of γ-benzyl-l-glutamate *N*-carboxyanhydride (BLG-NCA) using aminopropyl isobutyl POSS as the initiator; these hybrid polypeptides formed self-assembled nanoribbon structures in toluene [53]. Hayakawa et al. studied the microphase-separated structures of poly(styrene–*b*–methacrylate POSS) diblock copolymers, synthesized through anionic living polymerizations with well-defined molecular distributions [54,55]. Recently, Zhang et al. demonstrated that the incorporation of MIPOSS NPs could improve the thermal properties of poly(S-*alt*-MIPOSS) alternating copolymers [56].

**Scheme 1:**
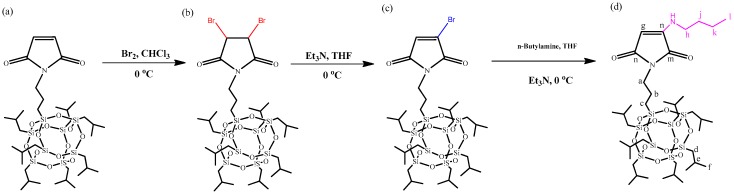
Chemical structures and synthesis of: (**a**) MIPOSS; (**b**) MIPOSS-2Br; (**c**) MIPOSS-Br; and (**d**) MIPOSS-NHBu.

In the present study, we synthesized maleimide/succinimide isobutyl POSS derivatives containing alkyl groups (Scheme 1). We then used 2-bromomaleimide and 2-aminomaleimide isobutyl POSS as raw materials to prepare several POSS-containing alternating copolymers—poly(S-*alt*-MIPOSS-Br), poly(S-*alt*-MIPOSS-NHBu), poly(AS-*alt*-MIPOSS-Br), and poly(AS-*alt*-MIPOSS-NHBu)—through simple free radical copolymerizations using azobisisobutyronitrile (AIBN) as the initiator in dry tetrahydrofuran (THF).

**Scheme 2:**
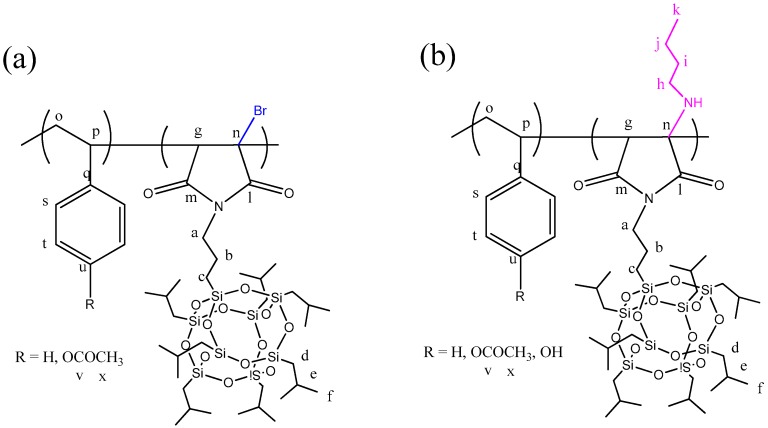
Chemical structures of: (**a**) poly(styrene-based-*alt*-MIPOSS-Br); and (**b**) poly(styrene-based-*alt*-MIPOSS-NHBu) copolymers.

Subsequently, we prepared a poly(HS-*alt*-MIPOSS-NHBu) alternating copolymer through acetoxyl hydrazinolysis of poly(AS-*alt*-MIPOSS-NHBu) in the presence of hydrazine monohydrate in 1,4-dioxane at room temperature (Scheme 2). We used ^1^H-, ^13^C-, and ^29^Si-NMR spectroscopy and Fourier transform infrared (FTIR) spectroscopy to confirm the structures of the monomers and polymers. Furthermore, we used differential scanning calorimetry (DSC) and thermogravimetric analysis (TGA) to examine the glass transition temperatures, thermal degradation temperatures, and char yields of the POSS-containing alternating copolymers. In addition, we used wide-angle X-ray diffraction (WAXD) and photoluminescence (PL) spectroscopy to determine the crystallinity and optical properties of these monomers and POSS-containing alternating polymers. PL spectroscopy also revealed the potential applications of MIPOSS-NHBu and poly(AS-*alt*-MIPOSS-NHBu) as sensors for metal ions in solution.

## 2. Experimental Section

### 2.1. Materials

Methanol (MeOH), *n*-butylamine, chloroform (CHCl_3_), dichloromethane (DCM), hexane, ethyl acetate (EA), tetrahydrofuran (THF), bromine (Br_2_), anhydrous magnesium sulfate (MgSO_4_), sodium chloride (NaCl), triethylamine (Et_3_N), and hydrazine monohydrate (N_2_H_4_·H_2_O) were purchased from Alfa-Aesar. Maleimide isobutyl POSS (MIPOSS) NPs was obtained from Hybrid Plastics and used without the purification. AIBN was purchased from Sigma-Aldrich (St. Louis, MO, USA), recrystallized from MeOH three times, and stored in a refrigerator to avoid decomposition. Styrene and 4-acetoxystyrene were also received from Sigma-Aldrich. Prior to free radical copolymerization, these monomers were passed through an aluminum oxide column to remove any inhibitor and then dried over CaH_2_ overnight under a N_2_ atmosphere. Zinc acetate dihydrate [Zn(CH_3_COO)_2_·2H_2_O], iron(III) nitrate nonahydrate [Fe(NO_3_)_3_·9H_2_O], iron(III) chloride (FeCl_3_), aluminum nitrate nonahydrate [Al(NO_3_)_3_·9H_2_O], and indium(III) chloride (InCl_3_) were purchased from Alfa-Aesar (Ward Hill, MA, USA). The poly(MIPOSS) homopolymer and the poly(S-*alt*-MIPOSS-Br) and poly(AS-*alt*-MIPOSS-Br) alternating copolymers were synthesized through free radical polymerizations according to a previous report [31].

*2,3-Dibromosuccinimide Isobutyl POSS (MIPOSS-2Br)* [57]. Br_2_ solution (0.65 g, 4.01 mmol) diluted in CHCl_3_ (5 mL) was added dropwise to a solution of maleimide isobutyl POSS (MIPOSS; 3.00 g, 3.15 mmol) in dry CHCl_3_ (20 mL) in an ice-bath under a N_2_ atmosphere. The mixture was then stirred for 24 h at room temperature. The solvent was evaporated under vacuum and the solid residue was extracted with DCM three times. The product was purified through column chromatography [SiO_2_; hexane/EA, 1:1 (*v*/*v*)] to obtain a white powder. ^1^H-NMR (500 MHz, CDCl_3_, δ, ppm) (Figure S1): 3.54 (N**CH_2_**CH_2_), 1.85 [**CH**(CH_3_)_2_], 1.68 (NCH_2_**CH_2_**), 0.97 [CH(**CH_3_**)_2_], 0.87 (Si**CH_2_**CH_2_CH_2_), 0.61 [Si**CH_2_**CH(CH_3_)_2_]. ^13^C-NMR (125 MHz, CDCl_3_, δ, ppm) (Figure S2): 177.58 (maleimide C=O), 41.6, 32.2, 29.3, 26.0, 25.7, 21.4, 9.4. FTIR (KBr, cm^−1^): 1778 (asymmetric imide C=O stretching), 1710 (symmetric imide C=O stretching), 2947–2888 (isobutyl CH stretch), 1226 (CN bending vibration), 1109 (Si–O–Si stretching of POSS core).

*2-Bromomaleimide Isobutyl POSS (MIPOSS-Br)* [57]. Et_3_N (0.150 g, 1.43 mmol) was added dropwise over 20 min to a solution of MIPOSS-2Br (1.50 g, 1.35 mmol) in dry THF (20 mL) in an ice bath under a N_2_ atmosphere. The mixture was then stirred for 72 h. The precipitate was filtered off and the solution concentrated (rotary evaporator) to afford a yellow solid. The crude product was purified through column chromatography (hexane:EA, 1:1) to obtain a yellow powder. ^1^H-NMR (500 MHz, CDCl_3_, δ, ppm): 6.69 (**CH**=C), 3.54 (N**CH_2_**CH_2_), 1.85 [**CH**(CH_3_)_2_], 1.68 (NCH_2_**CH_2_**), 0.97 [CH(**CH_3_**)_2_], 0.87 (Si**CH**_2_CH_2_CH_2_), 0.61 [Si**CH_2_**CH(CH_3_)_2_]. ^13^C-NMR (125 MHz, CDCl_3_, δ, ppm): 169.03, 166.72 (maleimide C=O), 134.67, 132.36, 46.31, 41.38, 38.44, 31.90, 9.01. FTIR (KBr, cm^−1^): 1768 (asymmetric imide C=O stretching), 1714 (symmetric imide C=O stretching), 1636 (C=C stretch), 2946–2887 (isobutyl CH stretch), 1238 (C–N bending vibration), 1109 (Si–O–Si stretching of POSS core).

**Figure 1:**
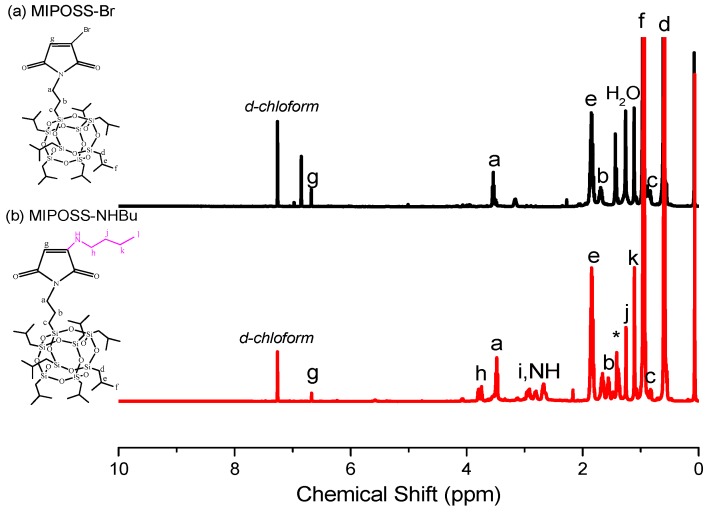
^1^H-NMR spectra of the monomers (**a**) MIPOSS-Br and (**b**) MIPOSS-NHBu.

**Figure 2:**
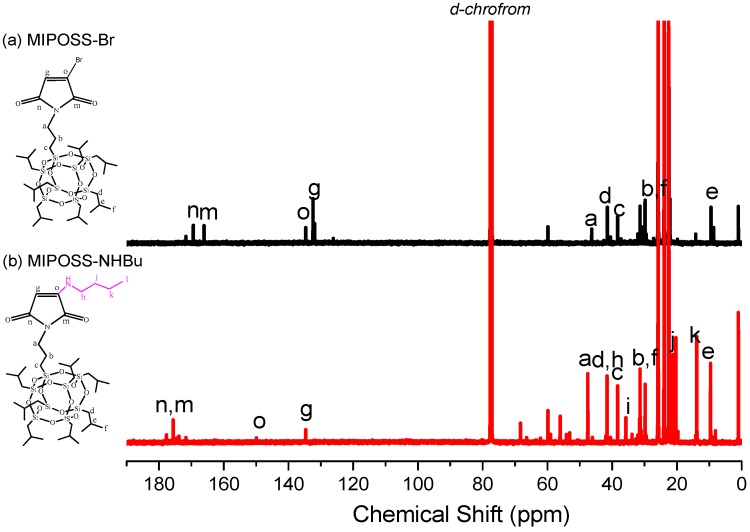
^13^C-NMR spectra of the monomers: (**a**) MIPOSS-Br; and (**b**) MIPOSS-NHBu.

**Figure 3:**
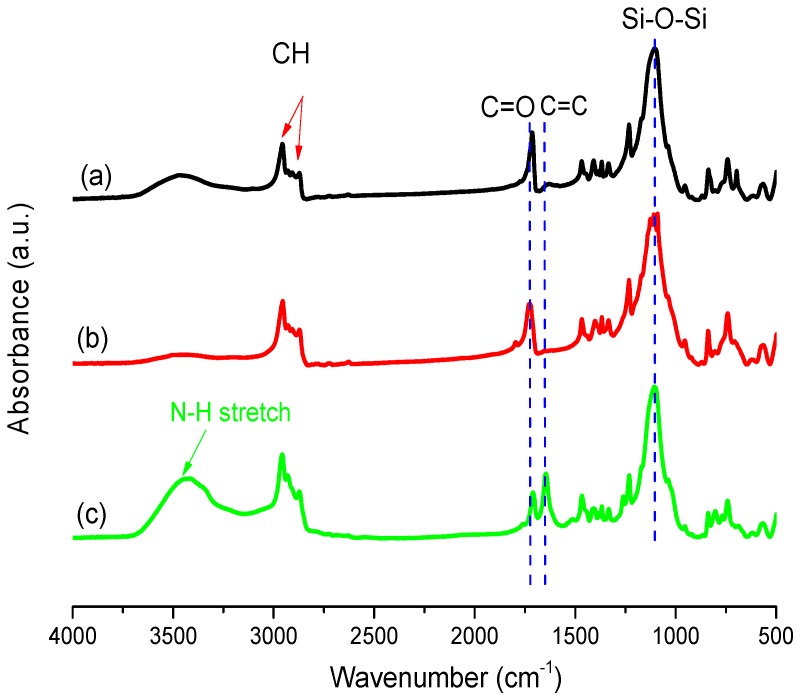
FTIR spectra of the monomers: (**a**) MIPOSS; (**b**) MIPOSS-Br; and (**c**) MIPOSS-NHBu.

*2-(Butylamino)-Maleimide Isobutyl POSS (MIPOSS-NHBu). n*-Butylamine (0.037 g, 0.50 mmol) was added to a solution of MIPOSS-Br (0.500 g, 0.484 mmol) in dry THF (20 mL) in an ice bath and then the mixture was stirred for 10 min. Et_3_N (0.052 g, 0.51 mmol) was added dropwise to the cooled reaction mixture (ice bath) and then the mixture was stirred for 24 h at room temperature. The solvent was evaporated under vacuum and the solid residue was extracted three times in with DCM (40 mL), water (20 mL), and aqueous NaCl (30 mL) and then dried over MgSO_4_. The crude mixture was purified through column chromatography (SiO_2_; hexane/EA, 1:1) to yield a yellow powder. ^1^H-NMR (500 MHz, CDCl_3_, δ, ppm) (Figure 1): 6.69 (**CH**=C), 3.54 (N**CH_2_**CH_2_), 3.95 (NH-**CH_2_**CH_2_), 1.85 [**CH**(CH_3_)_2_], 1.68 (NCH_2_**CH_2_**), 1.28 (NHCH_2_**CH_2_**), 1.08 (CH_2_CH_2_**CH_2_**CH_3_), 0.97 [CH(**CH_3_**)_2_] and [NH(CH_2_)_3_CH_3_], 0.87 (Si**CH_2_**CH_2_CH_2_), 0.61 [Si**CH_2_**CH(CH_3_)_2_]. ^13^C-NMR (125 MHz, CDCl_3_, δ, ppm) (Figure 2): 169.03, 166.72 (maleimide C=O), 134.67, 132.36, 46.31, 41.38, 38.44, 36.17, 20.45, 31.90, 14.58, 9.01. FTIR (KBr, cm^−1^) (Figure 3): 3444 (NH stretch), 1768 (asymmetric imide C=O stretching), 1714 (symmetric imide C=O stretching), 1636 (C=C stretch), 2946–2887 (isobutyl CH stretch), 1238 (C–N bending vibration), 1109 (Si–O–Si stretching of POSS core).

*Poly(S-alt-MIPOSS-Br), Poly(S-alt-MIPOSS-NHBu), and Poly(AS-alt-MIPOSS-NHBu) Alternating Copolymers.* A solution of MIPOSS-Br (0.50 g, 0.50 mmol) or MIPOSS-NHBu (0.40 g, 0.077 mmol), styrene (0.33 g, 3.16 mmol) or 4-acetoxystyrene (0.52 g, 3.17 mmol), and AIBN (5 wt %) were dissolved in dry THF (60 mL) was heated at 70–80 °C for 24 h. The copolymerization was quenched through cooling the flask in an ice bath and exposing the contents to air for 1 h. The crude polymer was precipitated upon pouring the reaction solution into a large amount of cold MeOH. The crude polymer was reprecipitated from cold THF/MeOH many times to remove any unreacted MIPOSS-Br or MIPOSS-NH and S or AS monomer. The alternating copolymers were dried at 50 °C under high vacuum overnight to remove any residual solvent. For poly(S-*alt*-MIPOSS-Br): ^1^H-NMR (500 MHz, CDCl_3_, δ, ppm): 7.04 (ArH in polystyrene backbone), 3.40 (polymer backbone and NCH_2_CH_2_), 1.85 [**CH**(CH_3_)_2_], 1.60 (Si**CH_2_**CH_2_), 0.96 [CH(**CH_3_**)_2_], 0.60 (Si**CH_2_**). ^13^C-NMR (125 MHz, CDCl_3_, δ, ppm): 178.4 (maleimide C=O), 169.7, 151.3, 130.6, 122.7, 41.7, 30.1, 9.9. FTIR (KBr, cm^−1^): 1773 (asymmetric C=O stretching, imide and C=O, OCOCH_3_ group), 1706 (symmetric imide C=O stretching), 1226 (C–N bending), 1109 (Si–O–Si stretching of POSS core). For poly(S-*alt*-MIPOSS-NHBu): ^1^H-NMR (500 MHz, CDCl_3_, δ, ppm): 7.04–6.57 (ArH in polystyrene backbone), 3.40 (polymer backbone and NC**H**_2_CH_2_), 2.50 (N**CH_2_**CH_2_), 2.12 (NCH_2_**CH_2_**), 1.85 [C**H**(CH_3_)_2_], 1.60 (SiC**H**_2_CH_2_), 1.13 (NCH_2_CH_2_**CH_2_**), 0.96 [CH(C**H**_3_)_2_] and (**CH_3_**CH_2_), 0.60 (SiC**H**_2_). ^13^C-NMR (125 MHz, CDCl_3_, δ, ppm): 171.32 (maleimide C=O), 128.6, 128.0, 126.2, 41.6, 32.2, 31.8, 29.3, 26.0, 25.7, 22.2, 21.4, 9.4. FTIR (KBr, cm^−1^): 1773 (asymmetric C=O stretching, imide and C=O, OCOCH_3_ group), 1706 (symmetric imide C=O stretching), 1226 (CN bending), 1109 (Si–O–Si stretching of POSS core). For poly(AS-*alt*-MIPOSS-NHBu), most of the signals in the ^1^H and ^13^C-NMR spectra were the same as those for poly(S-*alt*-MIPOSS-NHBu), with the following exceptions: ^1^H-NMR, 6.85–6.51 (ArH in polystyrene backbone), 2.26 (OCO**CH_3_**); ^13^C-NMR, 179.1 (C=O imide), 170.1 (C=O acetate), 29.77 ppm (**CH_3_**).

*Poly(HS-alt-MIPOSS-NHBu) Alternating Copolymer.* Hydrazine monohydrate (15.5 g, 3.10 mmol) was added dropwise over 1 h to a solution of poly(AS-*alt*-MIPOSS-NHBu) (0.50 g) in 1,4-dioxane (25 mL) in an ice bath. The mixture was then stirred at room temperature overnight. The solvent was evaporated under vacuum and the solid residue was dissolved in DCM (50 mL) and extracted with water to remove any excess hydrazine monohydrate. The combined organic phases were dried (MgSO_4_) for 1 h and then concentrated (rotary evaporator) to give a white powder. ^1^H-NMR (500 MHz, CDCl_3_, δ, ppm): 8.98 (OH), 7.05–6.46 (ArH in polystyrene backbone). ^13^C-NMR (125 MHz, CDCl_3_, δ, ppm): 179.42 (maleimide C=O), 155.86, 128.64, 114.84, 41.62, 32.21, 29.34, 26.07, 25.73, 22.20, 21.39, 9.45. FTIR (KBr, cm^−1^): 3380 (OH stretching), 2948–2888 (isobutyl group), 1776 (asymmetric C=O, imide), 1706 (symmetric C=O stretching, imide), 1226 (C–N bending), 1109 (Si–O–Si stretching of POSS core). Table 1 summarizes the number-average molecular weight (*M*_n_), the molecular weight distribution, and the thermal properties of these alternating copolymers.

### 2.2. Characterization

^1^H- and ^13^C-NMR spectra were recorded using an INOVA 500 spectrometer (McKinley Scientific, Sparta, NJ, USA), CDCl_3_ as the solvent, and tetramethylsilane (TMS) as the internal reference. A Bruker Tensor-27 FTIR spectrometer (Billerica, MA, USA) was used to quantitatively characterize the chemical structures of the alternating copolymers, which were cast from THF solutions onto KBr crystal plates. The spectra were collected from 32 scans at a resolution of 4 cm^−1^ at room temperature. The molecular weights and polydispersities of the synthesized alternating copolymers were determined using a Waters 510 gel permeation chromatography (GPC, Waters, Taipei, Taiwan) system equipped with a refractive index detector and three Ultrastyragel columns (100, 500, and 1000 Å) connected in series. DMF was the eluent, at a flow rate of 1 mL/min, at 40 °C. DSC measurements were performed using a TA Q-20 system (TA Instrument, New Castle, DE, USA), under N_2_ as a purge gas (50 mL/min), and at a heating rate of 20 °C/min. The sample (ca. 5–7 mg) was placed in a sealed aluminum sample pan. The thermal stabilities of the homopolymers and alternating copolymers were investigated using a TA Q-50 thermogravimetric analyzer (TA Instrument, New Castle, DE, USA), under N_2_ as a purge gas (60 mL/min), and at a heating rate of 20 °C/min from 30 to 800 °C. UV-vis spectra were recorded using a Shimadzu mini 1240 spectrophotometer (Shimadzu, Taipei, Taiwan). PL spectra were recorded at room temperature using a monochromatized Xe light source, solutions of polymers in THF at a concentration of 10^−4^ M, and an excitation wavelength of 330 nm. The quantum efficiencies (Φ_f_) in solution and in the solid state were measured using an integrated sphere (Ocean Optics). WAXD profiles were measured using the wiggler beamline BL17A1 of the National Synchrotron Radiation Research Center (NSRRC) of Hsinchu, Taiwan. A triangular bent Si (111) single crystal was used to obtain a monochromated beam having a wavelength (λ) of 1.32 Å. The samples were annealed prior to WAXD measurements.

## 3. Results and Discussion

### 3.1. Synthesis of MIPOSS-2Br, MIPOSS-Br, and MIPOSS-NHBu

In this study, observed unusually strong emissions from polymers containing butylamine maleimide isobutyl POSS NPs in the solid state and in solution and also investigated their application as metal ion sensors. First, we prepared MIPOSS-2Br through the reaction of MIPOSS with Br_2_ in dry CHCl_3_ at room temperature (Scheme 1b). A subsequent elimination reaction of MIPOSS-2Br with Et_3_N in dry THF afforded MIPOSS-Br (Scheme 1c), thereafter used as a raw material for the high-yield synthesis of MIPOSS-NHBu (Scheme 1d). The chemical structures of all of the monomers synthesized in this study were confirmed using ^1^H- and ^13^C-NMR and FTIR spectroscopies. Figure 1 displays the ^1^H-NMR spectra of MIPOSS-Br and MIPOSS-NHBu in CDCl_3_. The characteristic resonance of the **CH**=C units appeared as a signal at 6.69 ppm for both MIPOSS-Br and MIPOSS-NHBu. The signal at 3.54 ppm corresponded to the two protons of the N**CH_2_**CH_2_ unit, while the signal at 0.87 ppm corresponded to the two protons of the Si**CH_2_**CH_2_CH_2_ linkage between the maleimide ring and the POSS core. We assign the signals at 3.95 and 1.28 ppm to the NH**CH_2_**CH_2_ and NHCH_2_**CH_2_** units, respectively, of MIPOSS-NHBu (Figure 1b). Figure 2 presents the ^13^C-NMR spectra of MIPOSS-Br and MIPOSS-NHBu. The signal for the maleimide C=O groups appeared at 169.03 and 166.72 ppm. For all of the maleimide isobutyl POSS monomers, the signals of the CH=C group were centered at 132.25 and 134.67 ppm, while those at 46.07 and 9.46 ppm represented the resonances of the SiCH_2_CH_2_**C**H_2_N methylene unit and the SiCH_2_**C**H(CH_3_)_2_ methine unit, respectively, of the POSS core. The significant downfield shift for the signal of the CH=C group from 134.67 ppm MIPOSS-Br to 149.79 ppm for MIPOSS-NHBu confirmed the success of the substitution reaction.

Figure 3 presents the FTIR spectra of MIPOSS, MIPOSS-Br, and MIPOSS-NHBu. The characteristic absorption bands of the POSS core and the maleimide ring appeared at 2956–2878 cm^−1^ (aliphatic CH stretching); 1768 and 1714 cm^−1^ (asymmetric and symmetric imide C=O stretching); 1636 cm^−1^ (C=C stretching); 1238 cm^−1^ (C–N bending); and 1110 cm^−1^ (Si–O–Si stretching of POSS core); with a signal at 3445 cm^−1^ for N–H stretching of MIPOSS-NHBu appearing in Figure 3c. The NMR and FTIR spectra data indicated that we had successfully incorporated the alkyl chain in the maleimide isobutyl POSS NP unit.

### 3.2. Synthesis of Poly(S-alt-MIPOSS-Br), Poly(S-alt-MIPOSS-NHBu), Poly(AS-alt-MIPOSS-NBu), and Poly(HS-alt-MIPOSS-NBu)

As mentioned above, the mechanical properties, oxidation resistance, and thermal stability of polymers can all increase after the incorporation of POSS NPs into a polymer matrix to form organic/inorganic nanocomposites. Therefore, in this current study, we prepared several MIPOSS-NHBu fluorophore-containing alternating copolymers: poly(S-*alt*-MIPOSS-Br), poly(S-*alt*-MIPOSS-NHBu), poly(AS-*alt*-MIPOSS-NHBu), and poly(HS-*alt*-MIPOSS-NHBu). We confirmed the molecular structures of these MIPOSS-NHBu–containing copolymers through ^1^H-, ^13^C-, and ^29^Si-NMR spectroscopic, FTIR spectroscopic, and GPC analyses.

**Figure 4:**
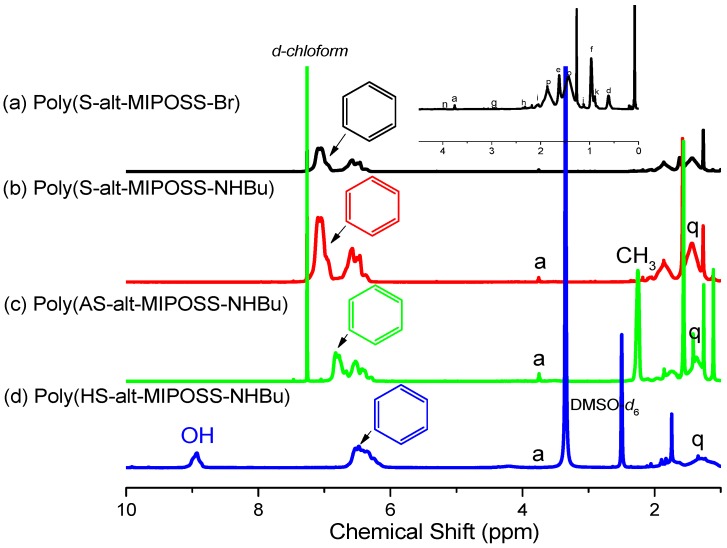
^1^H-NMR spectra of: (**a**) poly(S-*alt*-MIPOSS-Br); (**b**) poly(S-*alt*-MIPOSS-NHBu); (**c**) poly(AS-*alt*-MIPOSS-NHBu); and (**d**) poly(HS-*alt*-MIPOSS-NHBu).

Figure 4 displays the ^1^H-NMR spectra of these new alternating copolymers. The ^1^H-NMR spectrum of the poly(S-*alt*-MIPOSS-Br) copolymer (Figure 4a) features signals at 7.09–6.57 and 3.45 ppm representing the aromatic and side chain protons of MIPOSS and the methylene protons in the SiCH_2_CH_2_C**H**_2_N units. Other signals appeared at 1.82, 1.65, 0.94, and 0.60 ppm corresponding to the SiCH_2_C**H**(CH_3_)_2_ methine, SiCH_2_C**H**_2_CH_2_N methylene, SiCH_2_CH(C**H**_3_)_2_ methyl, and both the SiC**H**_2_CH_2_CH_2_N and SiC**H**_2_CH(CH_3_)_2_ methylene protons, respectively [40,42]. The ^1^H-NMR spectra of poly(S-*alt*-MIPOSS-NHBu), poly(AS-*alt*-MIPOSS-NHBu), and poly(HS-*alt*-MIPOSS-NHBu) (Figure 4b–d, respectively) feature their aromatic signals between 6.68 and 7.10 ppm. As displayed in Figure 4b for poly(S-*alt*-MIPOSS-NHBu), the characteristic proton signals were centered at 2.95, 2.20, 1.30, and 0.95 ppm, attributed to the NH**CH_2_**CH_2_, NHCH_2_**CH_2_**, NHCH_2_CH_2_C**H**_2_, and NHCH_2_CH_2_CH_2_**CH_3_** units, respectively. In addition, a peak appeared at 2.27 ppm in Figure 4c for poly(AS-*alt*-MIPOSS-NHBu), corresponding to the methyl unit in the acetyl groups (OCOC**H**_3_); this signal disappeared completely after hydrazine-mediated hydrolysis of the acetyl groups, affording poly(HS-*alt*-MIPOSS-NHBu) (Figure 4d), for which the signal of the **OH** groups appeared at 8.90 ppm [42]. Scheme 2 summarizes the other peak assignments.

**Figure 5:**
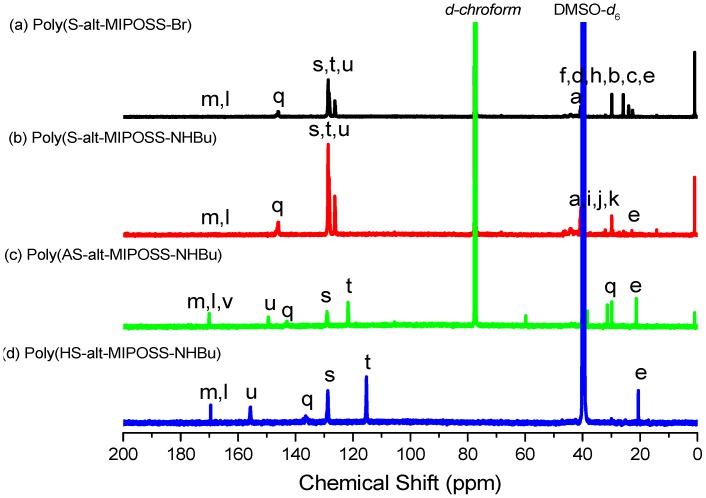
^13^C-NMR spectra of (**a**) poly(S-*alt*-MIPOSS-Br); (**b**) poly(S-*alt*-MIPOSS-NHBu); (**c**) poly(AS-*alt*-MIPOSS-NHBu); and (**d**) poly(HS-*alt*-MIPOSS-NHBu).

**Figure 6:**
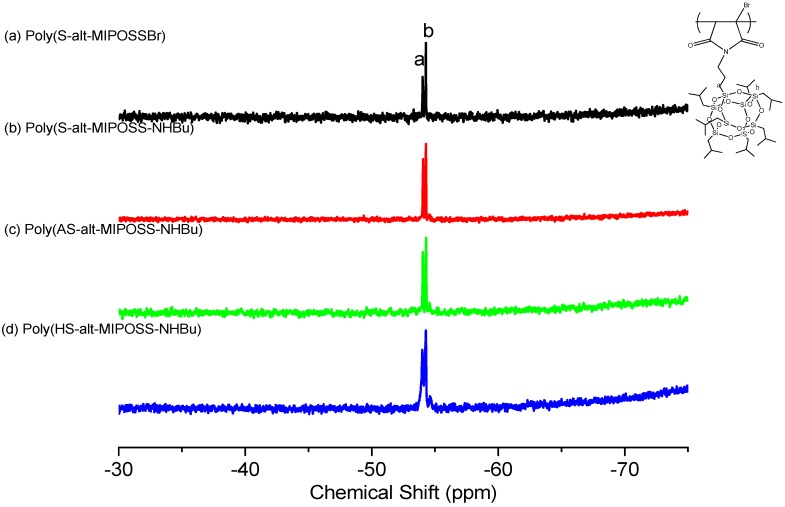
^29^Si-NMR spectra of: (**a**) poly(S-*alt*-MIPOSS-Br); (**b**) poly(S-*alt*-MIPOSS-NHBu); (**c**) poly(AS-*alt*-MIPOSS-NHBu); and (**d**) poly(HS-*alt*-MIPOSS-NHBu). Inset figure is corresponding to the chemical structure and peak assignment of ^29^Si NMR of poly(MIPOSS-Br).

Figure 5 presents the ^13^C-NMR spectra of these alternating copolymers in CDCl_3_ and DMSO-*d*_6_. The ^13^C-NMR spectra of poly(S-*alt*-MIPOSS-Br), poly(S-*alt*-MIPOSS-NHBu), and poly(AS-*alt*-MIPOSS-NHBu) (Figure 5a–c, respectively) feature signals at 177.96, 146.58, 145.89, 125.88, 41.28 and 9.01 ppm corresponding to the **C**=O groups, aromatic moieties, SiCH_2_CH_2_**C**H_2_N methylene units, and SiCH_2_**C**H(CH_3_)_2_ methine units, respectively. In Figure 5d, the signal from the aromatic rings at 155.87 (**Ph**OH, peak **u**) had shifted from 149.3 (**Ph**-OCOCH_3_) and 115.60 ppm (peak **t**) in Figure 5c; the absence of the signal for the Ph-OCO**C**H_3_ group at 31.20 ppm confirmed the successful hydrolysis of poly(HS-*alt*-MIPOSS-NHBu). Other peak assignment was also summarized in Scheme 2. We also recorded ^29^Si-NMR spectra of poly(S-*alt*-MIPOSS-Br), poly(S-*alt*-MIPOSS-NHBu), poly(AS-*alt*-MIPOSS-NHBu), and poly(HS-*alt*-MIPOSS-NHBu) to confirm the presence of their POSS cores and that no cage cleavage had occurred during the free radical copolymerizations of these alternating copolymers (Figure 6). Indeed, the ^29^Si-NMR spectra revealed signals at −54.03 (peak **a**) and −54.25 (peak **b**) ppm representing their O**Si**CH_2_CH_2_CH_2_N and O**Si**CH_2_CH(CH_3_)_2_ units, respectively.

Figure S3 displays the FTIR spectra of these alternating copolymers. Typical characteristic absorption bands for isobutyl CH stretching and Si–O–Si stretching of the maleimide isobutyl POSS structure in alternating copolymers appeared at 2920–2847 and 1105 cm^−1^, respectively, with the signal for the C=C bond at 1634 cm^−1^ disappearing, consistent with the occurrence of free radical copolymerization. The spectrum of poly(AS-*alt*-MIPOSS-NHBu) (Figure S3c) features a typical absorption band at 1768 cm^−1^ for C=O stretching of the acetyl groups in the AS units. This signal was absent in the spectrum of poly(HS-*alt*-MIPOSS-NHBu) (Figure S3d); instead, it displayed an absorption band at 3312 cm^−1^ for OH stretching in the HS units, confirming the successful hydrolysis of poly(HS-*alt*-MIPOSS-NHBu). The GPC profiles (Figure S4) of these new alternating copolymers displayed unimodal curves which were prepared via free radical polymerization process; Table 1 summarizes the data obtained for these alternating copolymers.

### 3.3. Thermal Properties of Monomers and Alternating Copolymers

**Figure 7:**
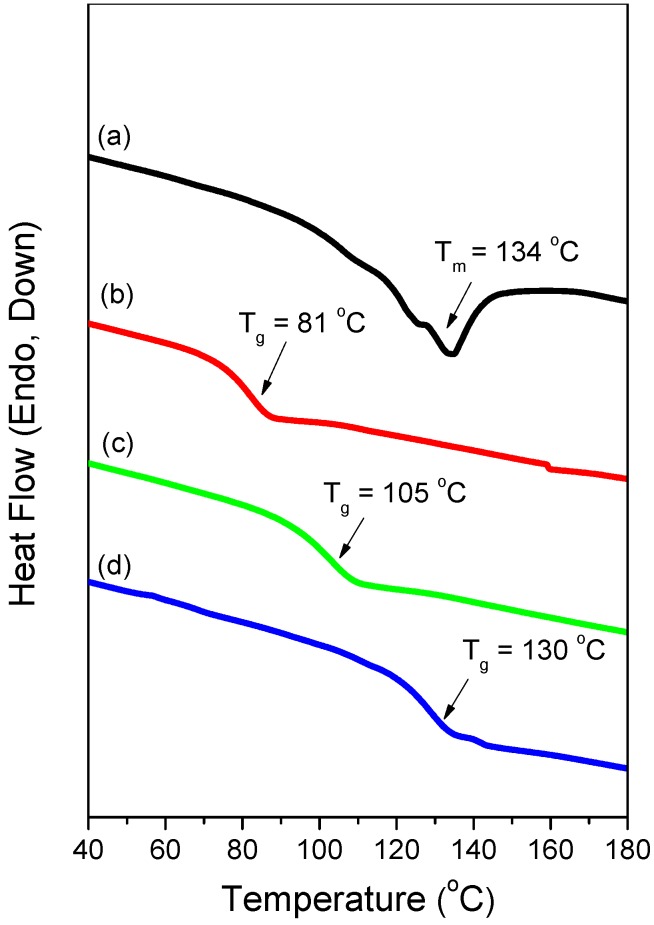
DSC thermograms of: (**a**) MIPOSS-NHBu; (**b**) poly(S-*alt*-MIPOSS-NHBu); (**c**) poly(AS-*alt*-MIPOSS-NHBu); and (**d**) poly(HS-*alt*-MIPOSS-NHBu).

We employed DSC under a N_2_ atmosphere to investigate the thermal properties of the monomers and alternating copolymers. Figure S5 presents the endothermic melting temperatures of MIPOSS, MIPOSS-2Br, MIPOSS-Br, and MIPOSS-NHBu. The melting temperature (*T*_m_) of the MIPOSS monomer was higher (171 °C) than those of MIPOSS-2Br (129 °C), MIPOSS-Br (168 °C), and MIPOSS-NHBu (118 °C), presumably because of the higher degrees of crystallinity and molecular packing of MIPOSS. Figure 7 presents DSC thermograms of the MIPOSS-NHBu monomer and the poly(S-*alt*-MIPOSS-NHBu), poly(AS-*alt*-MIPOSS-NHBu) and poly(HS-*alt*-MIPOSS-NHBu) copolymers, recorded under a N_2_ atmosphere. The DSC profile of the MIPOSS-NHBu monomer (Figure 7a) features an endothermic melting point near 118 °C, possibly representing aggregation or the crystalline structure of the POSS units. Lower crystallinity was evident for these three alternating copolymers, with the glass transition temperature (*T*_g_) of poly(HS-*alt*-MIPOSS-NHBu) (130 °C) higher than those of poly(AS-*alt*-MIPOSS-NHBu) (105 °C) and poly(S-*alt*-MIPOSS-NHBu) (81 °C), presumably because of intermolecular hydrogen bonding among the OH groups in the HS segments.

**Figure 8:**
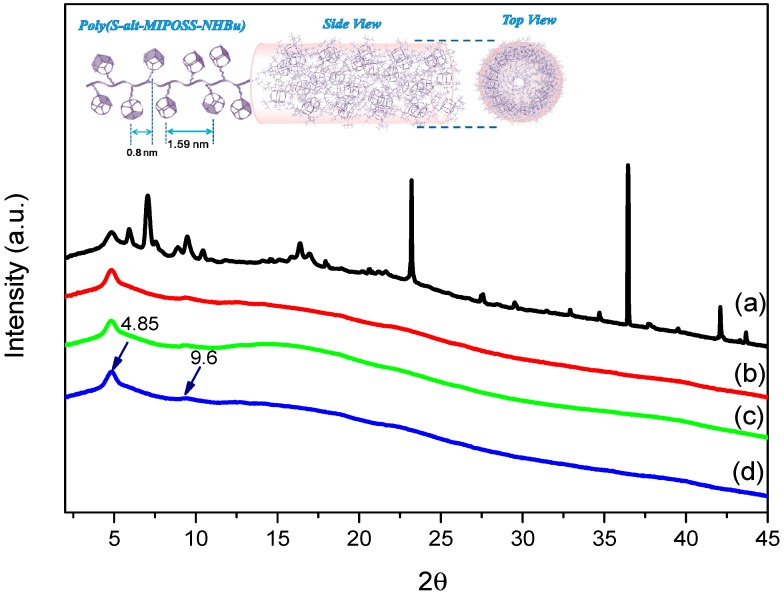
WAXD patterns of: (**a**) MIPOSS-NHBu; (**b**) poly(S-*alt*-MIPOSS-NHBu); (**c**) poly(AS-*alt*-MIPOSS-NHBu); and (**d**) poly(HS-*alt*-MIPOSS-NHBu). Inset Figure: the scheme about the *d*-spacing of poly(S-*alt*-MIPOSS-NHBu) alternating copolymer.

We used TGA under a N_2_ atmosphere to determine the decomposition temperatures and char yields of these alternating copolymers (Figure S6). The degradation temperatures and char yields of poly(S-*alt*-MIPOSS-Br) and poly(S-*alt*-MIPOSS-NHBu) were higher than those of the standard PS homopolymer (337 °C and 0%, respectively) because of the steric bulk of the rigid-cage MIPOSS units and the readier pyrolysis of the PS main chain [51,52]. The degradation temperature and char yield of alkylamine-functionalized poly(S-*alt*-MIPOSS-NHBu) (355 °C and 6.9%, respectively) were lower than those of the bromine-functionalized poly(S-*alt*-MIPOSS-Br) (364 °C and 12.2%, respectively); this behavior was expected because an alkyl chain possesses thermal stability lower than that of a bromide group. To examine the crystalline properties of the synthesized maleimide POSS derivatives and the amorphous characteristics of the alternating copolymers, we performed WAXD analyses at room temperature (Figure 8). The WAXD spectrum in Figure 8a features four diffraction peaks at 6.97 (101), 9.40 (110), 10.39 (102) and 16.29 (113) that correspond to the rhombohedral crystal structure of the MIPOSS unit (Figure S7). After introduction of S, AS, and HS units as inert diluent segments for the MIPOSS-NHBu units in poly(S-*alt*-MIPOSS-NHBu), poly(AS-*alt*-MIPOSS-NHBu), and poly(HS-*alt*-MIPOSS-NHBu), the WAXD profiles featured only broad amorphous halos and revealed the destruction of the crystalline structure of the MIPOSS unit (Figure 8b–d). The spectra of all the alternating copolymers exhibited two major sharp diffraction peaks at values of 2θ of 4.85° and 9.6°, representing *d*-spacing of 1.59 and 0.80 nm, respectively [55]; the first peak is consistent with the average distance between the POSS cages in the MIPOSS segment, while the other is due to the average distance between the maleimide groups of the MI-POSS segments [55]. The formed *d*-spacing is larger than that found for the pure PMAPOSS segment (*d* = 1.0 and 0.49 nm) [58], presumably because the functional NHBu groups on the main chain and inserted styrene-based segments into the MIPOSS-NHBu units expanded the *d*-spacing of these alternating copolymers, as displayed in inset scheme of Figure 8. Herein, we also observed that the full-width at half maximum of these two peaks did not change, indicating that the particle size of POSS did not change after incorporation after introduction of S, AS, and HS units into MIPOSS-NHBu segment. Thus, the maleimide monomer and the alternating copolymers possessed crystalline and amorphous structures, respectively, consistent with the results of the DSC analyses in Figure 7.

### 3.4. Optical Properties

**Figure 9:**
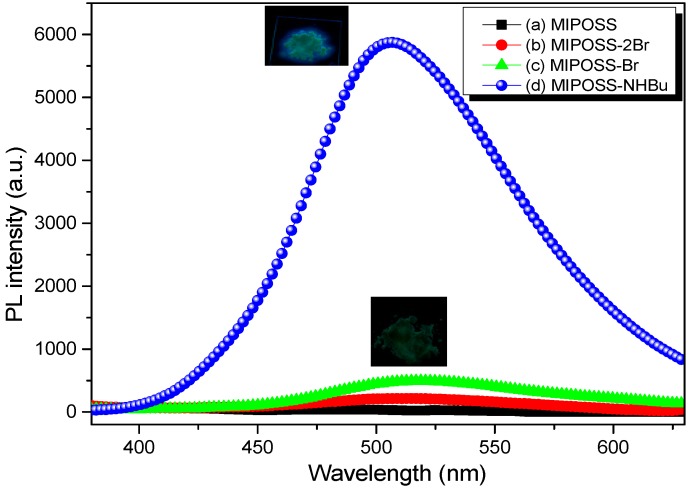
PL spectra of: (**a**) MIPOSS; (**b**) MIPOSS-2Br; (**c**) MIPOSS-Br; and (**d**) MIPOSS-NHBu, in the solid state (excitation: 330 nm). Top insert figure is photo image for MIPOSS-NHBu and the bottom insert figure is photo image for MIPOSS-Br under excitation at 330 nm.

Next, we studied the relationship between the molecular structure and photophysical properties in solid state and solution state for the series of maleimide and succinimide isobutyl POSS derivatives containing 2-butylamino units. Figure S8 shows the UV-vis spectra of MIPOSS, MIPOSS-2Br, MIPOSS-Br and MIPOSS-NHBu in dichloromethane solution (10^−4^ M). These monomers exhibited absorptions peaks at 234, 235, 245 and 236 nm, respectively which can be attributed to a π–π* transitions. Figure 9 displays the PL spectra of maleimide and succinimide isobutyl POSS in the solid state. Interestingly, MIPOSS-NHBu display an unusual fluorescence, with a strong emission peak near 506.6 nm, resulting from the spatial separation of the highest occupied molecular orbital (HOMO) and lowest unoccupied molecular orbital (LUMO), based on density functional theory calculations as reported in previous literature [34]. In contrast, MIPOSS-2Br and MIPOSS-Br did not show any emission peak, presumably because of the internal heavy atom effects of their bromine atoms. Figure S10 displays the UV-vis absorption spectra of poly(S-*alt*-MIPOSS-Br), poly(S-*alt*-MIPOSS-NHBu), poly(AS-*alt*-MIPOSS-Br), and poly(AS-*alt*-MIPOSS-NHBu) in dichloromethane solution (10^−4^ M). These alternating copolymers showed absorption peaks at 259, 258, 257 and 243, 264 nm, respectively which can be due to a π–π* and n–π* transitions.

**Figure 10:**
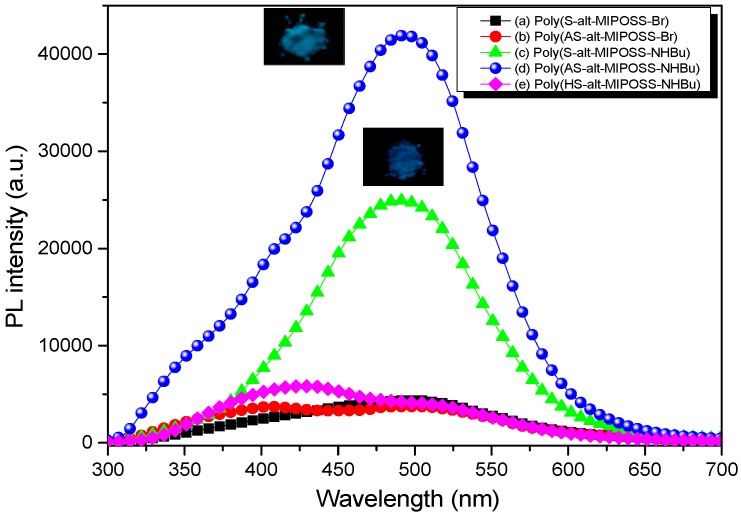
PL spectra of: (**a**) poly(S-*alt*-MIPOSS-Br); (**b**) poly(AS-*alt*-MIPOSS-Br) MIPOSS; (**c**) poly(S-*alt*-MIPOSS-NHBu; (**d**) poly(AS-*alt*-MIPOSS-NHBu); and (**e**) poly(HS-*alt*-MIPOSS-NHBu), in the solid state (excitation: 330 nm). Top insert figure is photo image for poly(AS-*alt*-MIPOSS-NHBu) and the bottom insert figure is photo image for poly(S-*alt*-MIPOSS-NHBu) under excitation at 330 nm.

Figure 10 presents the PL spectra of poly(S-*alt*-MIPOSS-Br), poly(AS-*alt*-MIPOSS-Br), poly(S-*alt*-MIPOSS-NHBu), poly(AS-*alt*-MIPOSS-NHBu), and poly(HS-*alt*-MIPOSS-NHBu) in the solid state, with excitation at 330 nm. The spectra of the oxygenic non-conjugated poly(S-*alt*-MIPOSS-NHBu) and poly(AS-*alt*-MIPOSS-NHBu) both featured strong emission peaks at 492.3 and 493.3 nm, presumably because of the higher degrees of clustering of their locked C=O groups. In contrast, the spectrum of poly(HS-*alt*-MIPOSS-NHBu) featured two weak fluorescence signals of low intensity at 419.0 and 506.2 nm, resulting from the π–π interactions of the phenyl rings and C=O groups in the poly(MIPOSS) segments. The unexpected strong fluorescence of poly(AS-*alt*-MIPOSS-NHBu) in the solid state, relative to those of the other alternating copolymers, was presumably caused by: (i) strong dipole–dipole interactions between AS**···**AS and MIPOSS**···**MIPOSS units; and (ii) strong intermolecular hydrogen bonding between the NH groups from the MIPOSS-NHBu segments and the C=O groups from the AS segments; together, these effects hindered the free rotation of the copolymer chains about the C–C single bonds and, thereby, enhanced the emission of poly(AS-*alt*-MIPOSS-NHBu). The measured quantum efficiencies (Ф_f_) of poly(S-*alt*-MIPOSS-NHBu), poly(AS-*alt*-MIPOSS-NHBu), and poly(HS-*alt*-MIPOSS-NHBu) in the solid state were 78.8%, 93.8%, and 31.4%, respectively. Compared with poly(S-*alt*-MIPOSS), poly(S-*alt*-MIPOSS-NHBu) exhibited its unexpectedly strong emission enhancement because the 2-amino-succinimide unit underwent intramolecular hydrogen bonding with the C=O groups of the MIPOSS units to lock the C=O groups in the MIPOSS units.

**Figure 11:**
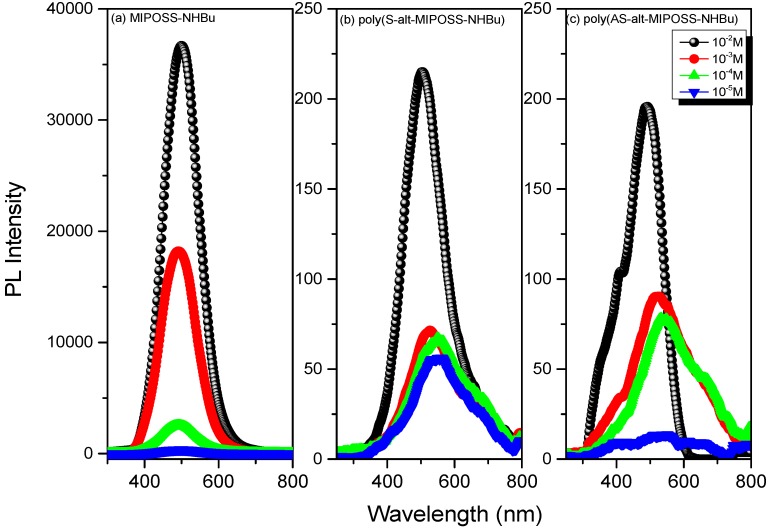
Fluorescence spectra of: (**a**) MIPOSS-NHBu monomer; (**b**) poly(S-*alt*-MIPOSS-NHBu); and (**c**) poly(AS-*alt*-MIPOSS-NHBu), in THF at concentrations from 10^−5^ to 10^−2^ M (excitation: 330 nm).

Figure 11 presents the PL spectra of MIPOSS-NHBu, poly(S-*alt*-MIPOSS-NHBu) and poly(AS-*alt*-MIPOSS-NHBu) as solutions in THF at various concentrations, with excitation at 330 nm. Upon increasing the concentrations of the monomer and polymers (from 10^−5^ to 10^−2^ M) in THF (a good solvent), the emission intensities increased. On the other hand, the fluorescence intensities of these oxygenic nonconjugated components decreased in dilute solutions. These results suggest that these materials may be AIE materials. The MIPOSS-NHBu monomer has good solubility in hexane, DCM, 1,4-dioxane, acetone, EA, and DMSO; poly(S-*alt*-MIPOSS-NHBu) was soluble in DCM, 1,4-dioxane, and acetone. We investigated the effects of polar/nonpolar solvents and the environment on the emission behavior of MIPOSS-NHBu and poly(S-*alt*-MIPOSS-NHBu). The nitrogen atom of the NH group is an electron donor and the C=O group in MIPOSS-NHBu is an electron acceptor. Thus, we might expect MIPOSS-NHBu and poly(S-*alt*-MIPOSS-NHBu) to be highly sensitive to solvents because electron transfer should occur from the N atoms to the C=O groups in the imide units.

**Figure 12:**
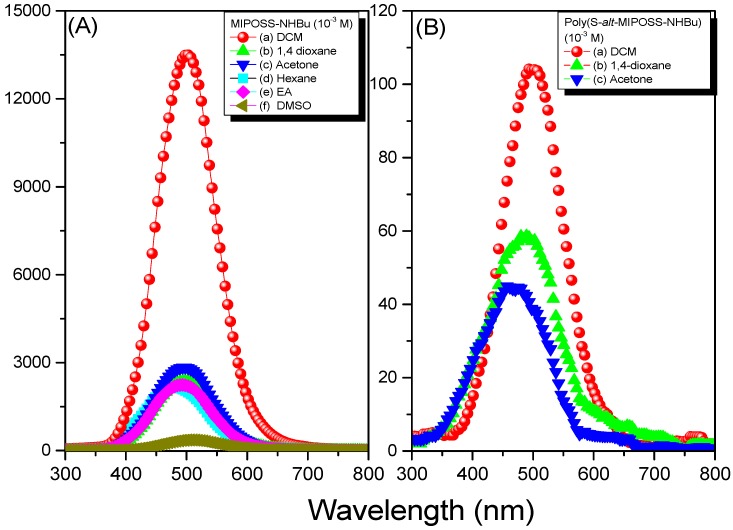
Fluorescence spectra of the: (**A**) monomer MIPOSS-NHBu; and (**B**) copolymer poly(S-*alt*-MIPOSS-NHBu), in various solvents at a concentration of 10^−4^ M (excitation: 330 nm).

**Figure 13:**
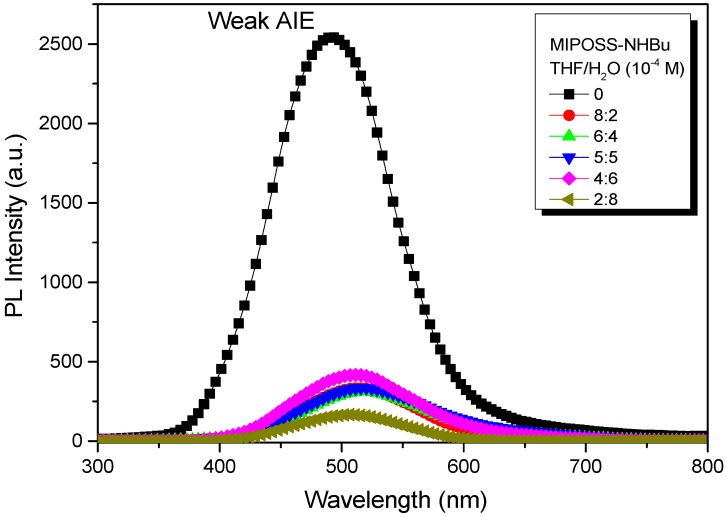
Fluorescence spectra of the monomer MIPOSS-NHBu, in THF/H_2_O at various ratios at a concentration of 10^−4^ M (excitation: 330 nm).

Figure 12 reveals that both MIPOSS-NHBu and poly(S-*alt*-MIPOSS-NHBu) have high fluorescence intensities in DCM. In the aprotic solvents acetone and DMSO, the PL intensities of MIPOSS-NHBu and poly(S-*alt*-MIPOSS-NHBu) decreased significantly, due to the formation of hydrogen bonds between the NH groups in the MIPOSS-NHBu units and the S=O (in DMSO) and C=O (in acetone) groups, and also intramolecular charge transfer that weakened the intramolecular hydrogen bonding with the C=O groups of MIPOSS. Based on the concentration-enhanced emissions in Figure 11, we predicted that MIPOSS-NHBu, poly(S-*alt*-MIPOSS-NHBu), and poly(AS-*alt*-MIPOSS-NHBu) would be AIE materials. To confirm our hypothesis, we recorded PL spectra of MIPOSS-NHBu, poly(S-*alt*-MIPOSS-NHBu), and poly(AS-*alt*-MIPOSS-NHBu) in THF/H_2_O mixtures as a solvent pair. For MIPOSS-NHBu, upon increasing the H_2_O content from 0% to 50% (Figure 13 and Figure S9), the emission intensities decreased dramatically; the emission was enhanced again when the H_2_O content was 60%, but did not return to the original level. Similar phenomena occurred for poly(S-*alt*-MIPOSS-NHBu) and poly(AS-*alt*-MIPOSS-NHBu) in THF/H_2_O mixtures (Figure S11).

The PL intensities of poly(S-*alt*-MIPOSS-NHBu) and poly(AS-*alt*-MIPOSS-NHBu) increased upon increasing the H_2_O content from 40% to 50%. We assign the decreases in the emission intensities of MIPOSS-NHBu, poly(S-*alt*-MIPOSS-NHBu), and poly(AS-*alt*-MIPOSS-NHBu) to the high polarity of H_2_O; this behavior generally occurs in donor–acceptor luminogens. Therefore, MIPOSS-NHBu, poly(S-*alt*-MIPOSS-NHBu), and poly(AS-*alt*-MIPOSS-NHBu) displayed weak AIEE activity in solution. We also measured the quantum efficiencies (Ф_f_) of maleimide, succinimide isobutyl POSS, and the novel alternating copolymers in the solid state and in solution; Table S1 summarizes the values. Furthermore, we used dynamic light scattering (DLS) to measure the sizes of the particles of MIPOSS-NHBu, poly(S-*alt*-MIPOSS-NHBu), and poly(AS-*alt*-MIPOSS-NHBu) in the THF/H_2_O mixtures (Figures S12 and S13). Upon increasing the H_2_O content from 40% to 50% to 60%, the sizes of nanoaggregate structures formed from MIPOSS-NHBu decreased from 273 to 161 to 57 nm (Figure S12). Upon increasing the H_2_O content from 40, 50, 60 and 80, the sizes of NPs for poly(S-*alt*-MIPOSS-NHBu) were 886, 423, 303 and 187 nm, respectively, while for poly(AS-*alt*-MIPOSS-NHBu) they were 1102, 808, 724 and 255 nm, respectively (Figure 13).

### 3.5. Fluorescence Responses of MIPOSS-NHBu and Poly(AS-alt-MIPOSS-NHBu) toward Metal Ions

**Figure 14:**
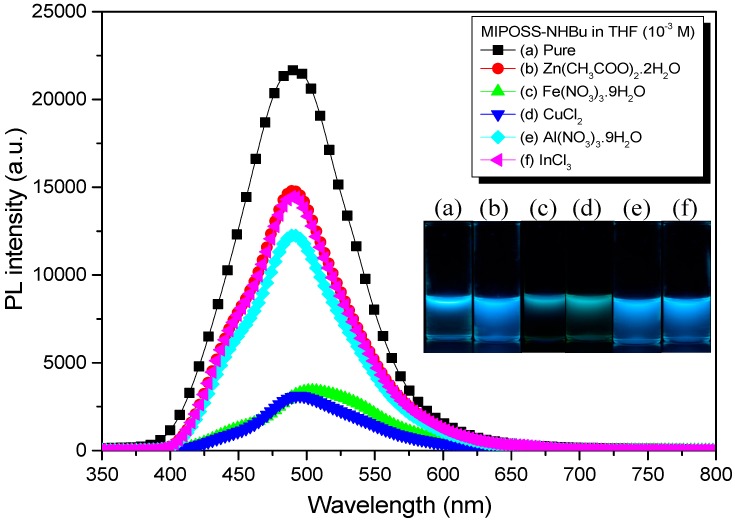
Fluorescence spectra (excitation: 330 nm) of the monomer MIPOSS-NHBu (concentration: 10^−3^ M) in the presence of various metal ions: (**a**) pure MIPOSS-NHBu; (**b**) Zn(CH_3_COO)_2_·2H_2_O; (**c**) Fe(NO_3_)_2_·9H_2_O; (**d**) CuCl_2_; (**e**) Al(NO_3_)_3_·9H_2_O; and (**f**) InCl_3_.

**Figure 15:**
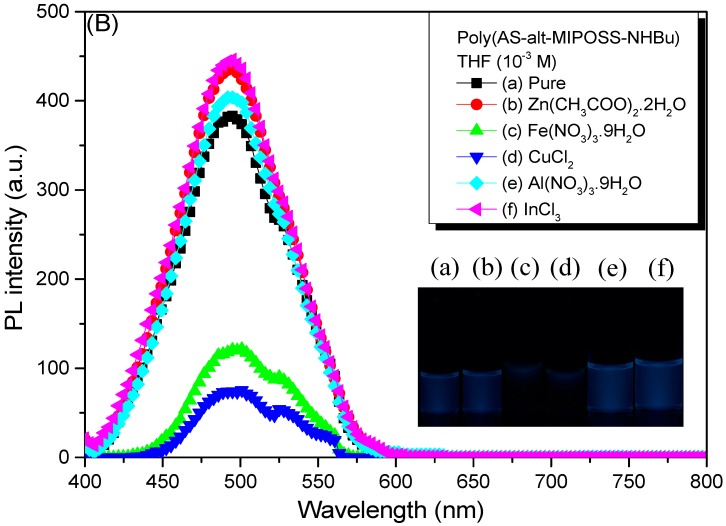
Fluorescence spectra (excitation: 330 nm) of the copolymer poly(AS-*alt*-MIPOSS-NHBu) (concentration: 10^−3^ M) in the presence of various metal ions: (**a**) pure MIPOSS-NHBu; (**b**) Zn(CH_3_COO)_2_·2H_2_O; (**c**) Fe(NO_3_)_2_·9H_2_O; (**d**) CuCl_2_; (**e**) Al(NO_3_)_3_·9H_2_O; and (**f**) InCl_3_.

We examined the selectivity response of solutions of MIPOSS-NHBu and poly(AS-*alt*-MIPOSS-NHBu) in THF (10^−3^ M) to five different metal cations: Zn^2+^, Fe^3+^, Cu^2+^, Al^3+^, and In^3+^. For MIPOSS-NHBu, the PL emission intensity decreased slightly upon the addition Zn^2+^, Al^3+^, and In^3+^, relative to the original state, but it decreased significantly upon the addition of Fe^3+^ and Cu^2+^ (Figure 14). Poly(AS-*alt*-MIPOSS-NHBu) (Figure 15) exhibited similar phenomena, presumably because of the formation of strong Fe^3+^ and Cu^2+^ complexes with the C=O groups in the imide units and with the NH groups, through metal–ligand interactions.

## 4. Conclusions

We have synthesized the monomer MIPOSS-NHBu and the alternating copolymers poly(S-*alt*-MIPOSS-NHBu), poly(AS-*alt*-MIPOSS-NHBu), and poly(AS-*alt*-MIPOSS-NHBu) through free radical copolymerizations, with their structures confirmed using NMR and FTIR spectroscopy. Thermal analyses revealed that the thermal stabilities and char yields of the POSS-containing alternating copolymers improved after incorporating the inorganic MIPOSS units. WAXD analyses indicated that these POSS-containing alternating polymers were amorphous. Because of intramolecular hydrogen bonding between the amino and dihydrofuran-2,5-dione groups and the clustering of locked C=O groups from the POSS NPs of the MIPOSS-NHBu units, the intramolecular motion of the polymer chain was restricted, thereby resulting in stronger light emission than that from the MIPOSS unit. These novel luminescent copolymers displayed good selectivity responses for the detection of metal ions.

## Figures and Tables

**Table 1:** Molecular weights, glass transition temperatures, and quantum yields of the alternating copolymers.

Sample	*M*_n_^a^	*M*_w_^a^	PDI ^a^	*T*_g_^b^ (°C)	*T*_d_^c^ (°C)	Quantum Yield ^d^ (%)
S-*alt*-MIPOSS-Br	23,900	40,000	1.67	83	364	52.4
S-*alt*-MIPOSS-NHBu	10,900	18,964	1.74	81	355	78.8
AS-*alt*-MIPOSS-Br	74,200	106,200	1.43	119	356	63.2
AS-*alt*-MIPOSS-NHBu	26,800	46,000	1.71	105	326	93.8
HS-*alt*-MIPOSS-NHBu	65,300	95,900	1.46	130	320	31.4

^a^ Measured through GPC analysis; ^b^ Measured through DSC; ^c^ Measured through TGA; ^d^ Measured using an integrated sphere (Ocean Optics).

## Supplementary Materials

The following are available online at www.mdpi.com/2073-4360/9/3/103/s1. Table S1: Quantum yield (%) of MIPOSS-NHBu, and poly(S-*alt*-MIPOSS-Br). Figure S1: 1H NMR spectrum of MIPOSS-2Br. Figure S2: 13C NMR spectrum of MIPOSS-2Br. Figure S3: FTIR spectra of (a) poly(S-*alt*-MIPOSS-NHBu), (b) poly(AS-*alt*-MIPOSS-NHBu), and (c) poly(HS-*alt*-MIPOSS-NHBu). Figure S4: GPC analyses of the alternating copolymers (a) poly(S-*alt*-MIPOSS-Br), (b) poly(AS-*alt*-MIPOSS-Br), (c) poly(S-*alt*-MIPOSS-NHBu), and (d) poly(AS-*alt*-MIPOSS-NHBu). Figure S5: DSC thermograms of (a) MIPOSS, (b) MIPOSS-2Br, (c) MIPOSS-Br, and (d) MIPOSS-NHBu. Figure S6: TGA analyses of the alternating copolymers poly(S-*alt*-MIPOSS-Br) and poly(S-*alt*-MIPOSS-NHBu). Figure S7: WAXD patterns of the monomer MIPOSS. Figure S8: UV-vis spectra of the monomers (a) MIPOSS, (b) MIPOSS-2Br, (c) MIPOSS-Br and (d) MIPOSS-NHBu in DCM solution (10–4 M). Figure S9: Fluorescence spectra of the monomer MIPOSS-NHBu in THF solution at concentrations from 10–5 to 10–2 M (excitation: 330 nm). Figure S10: UV-vis spectra of the alternating copolymers (a) poly(S-*alt*-MIPOSS-Br), (b) poly(S-*alt*-MIPOSS-NHBu), (c) poly(AS-*alt*-MIPOSS-Br), and (d) poly(AS-*alt*-MIPOSS-NHBu) in DCM solution (10–4 M). Figure S11: Fluorescence spectra (excitation: 330 nm) of (A) poly(S-*alt*-MIPOSS-NHBu) and (B) poly(AS-*alt*-MIPOSS-NHBu) in THF/H2O at various ratios, at a concentration of 10–4 M. Figure S12: DLS analysis of the monomer MIPOSS-NHBu in THF/H2O at various ratios, at a concentration of 10–4 M. Figure S13: DLS analysis of (A) poly(S-*alt*-MIPOSS-NHBu) and (B) poly(AS-*alt*-MIPOSS-NHBu) in THF/H2O at various ratios, at a concentration of 10–4 M.
