# Supplementary material for: Unusual Emission of Polystyrene-Based Alternating Copolymers Incorporating Aminobutyl Maleimide Fluorophore-Containing Polyhedral Oligomeric Silsesquioxane Nanoparticles
Source: Polymers (Basel). 2017 Mar 15;9(3):103. doi: 10.3390/polym9030103 (PMC6432138; doi:10.3390/polym9030103)
Supplement: Supplementary file 1 [file polymers-09-00103-s001.pdf]

Supplementary Materials: Unusual Emission of Polystyrene-Based Alternating Copolymers from Aminobutyl Maleimide Fluorophore-Containing Polyhedral Oligomeric Silsesquioxanes Nanoparticle

Mohamed Gamal Mohamed, Yu-Ru Jheng, Shu-Ling Yeh, Tao Chen and Shiao-Wei Kuo

Table S1. Quantum yield (%) of MIPOSS-NHBu, and poly(S-*alt*-MIPOSS-Br).

Sample	DCM	Dioxane	Acetone	DMSO	EA	Hexane
MIPOSS-NHBu	65.8	11.8	12.7	1.9	10.3	10.5
S-*alt*-MIPOSS-NHBu	13.3	6.9	1.4	-	-	-

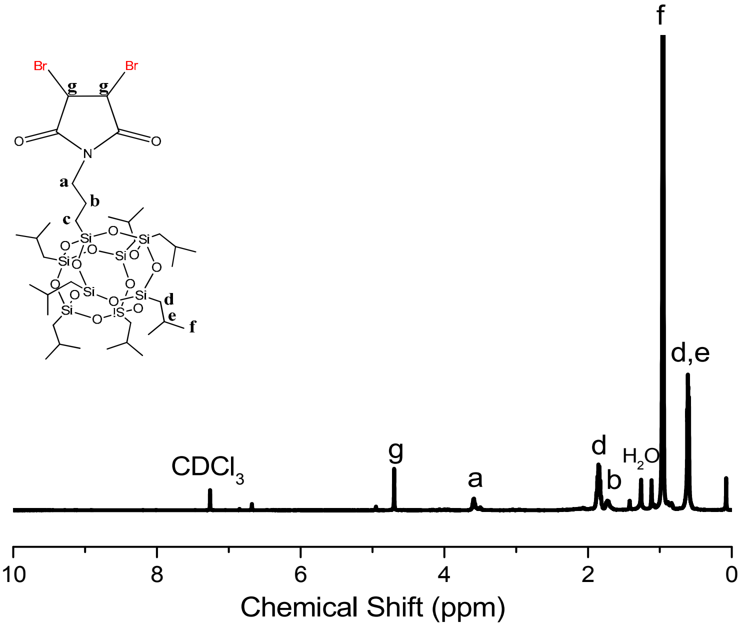

Figure S1. ^{1}H NMR spectrum of MIPOSS-2Br.

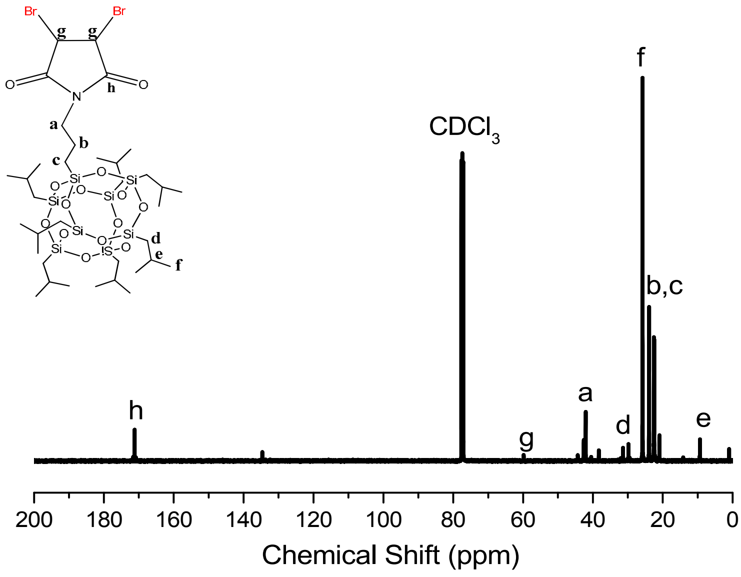

Figure S2. ^{13}C NMR spectrum of MIPOSS-2Br.

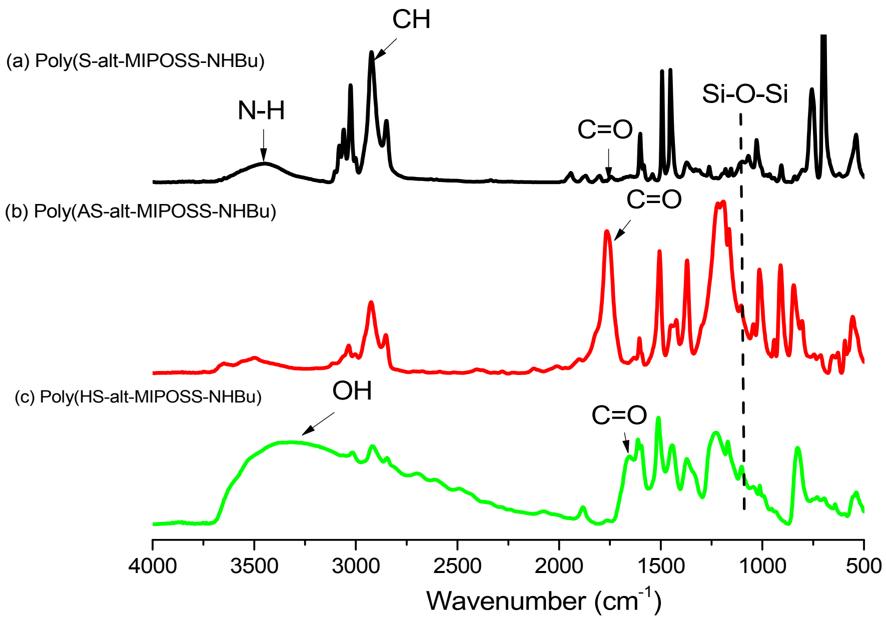

Figure S3. FTIR spectra of (a) poly(S-*alt*-MIPOSS-NHBu), (b) poly(AS-*alt*-MIPOSS-NHBu), and (c) poly(HS-*alt*-MIPOSS-NHBu).

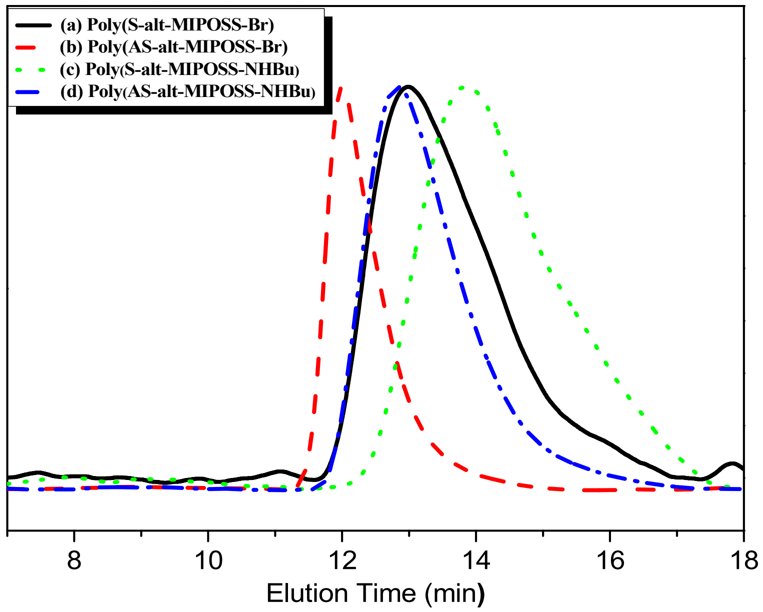

Figure S4. GPC analyses of the alternating copolymers (a) poly(S-*alt*-MIPOSS-Br), (b) poly(AS-*alt*-MIPOSS-Br), (c) poly(S-*alt*-MIPOSS-NHBu), and (d) poly(AS-*alt*-MIPOSS-NHBu).

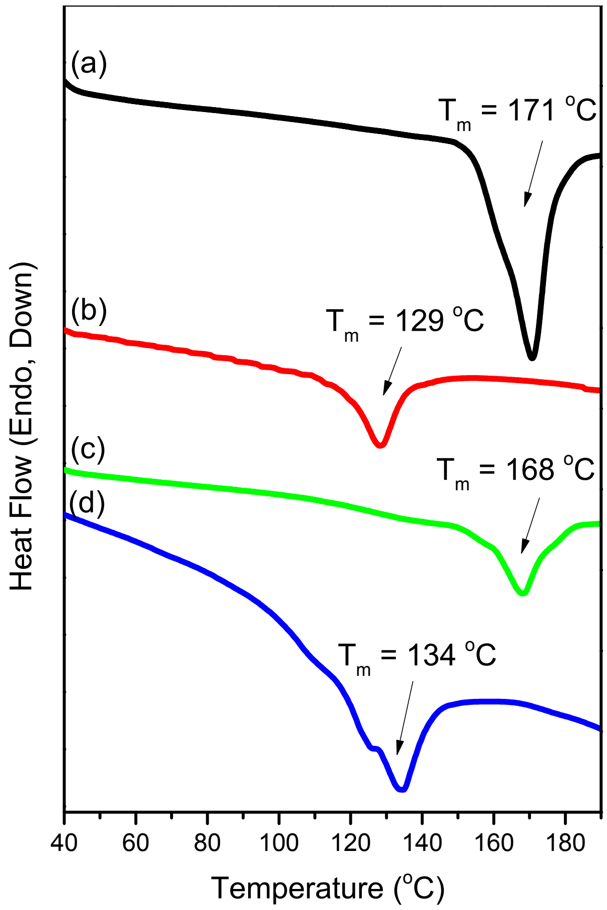

Figure S5. DSC thermograms of (a) MIPOSS, (b) MIPOSS-2Br, (c) MIPOSS-Br, and (d) MIPOSS-NHBu.

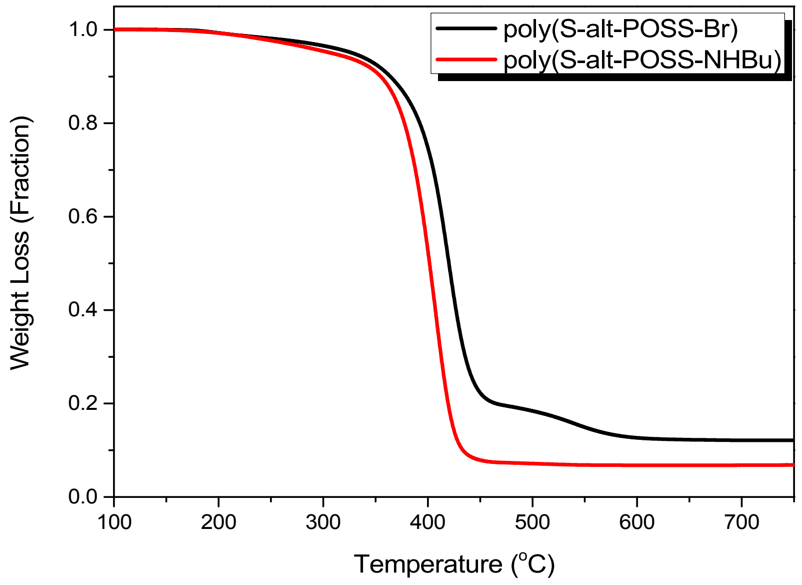

Figure S6. TGA analyses of the alternating copolymers poly(S-*alt*-MIPOSS-Br) and poly(S-*alt*-MIPOSS-NHBu).

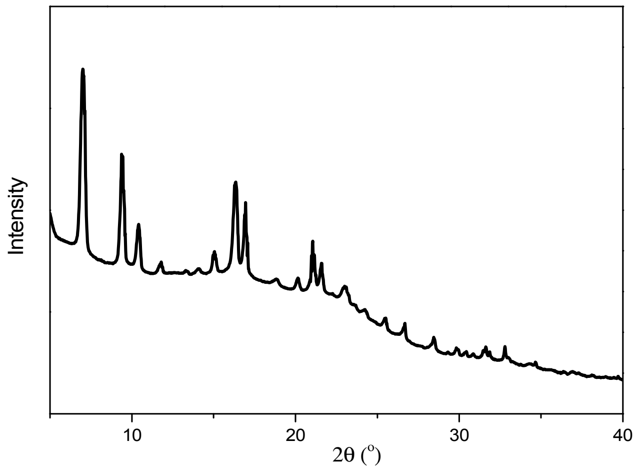

Figure S7. WAXD patterns of the monomer MIPOSS.

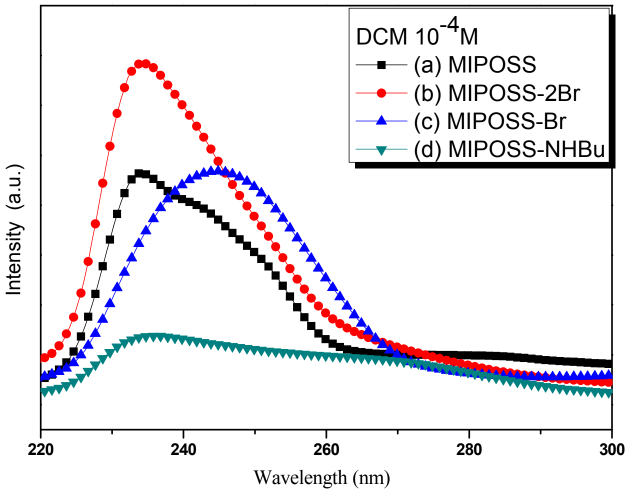

Figure S8. UV-vis spectra of the monomers (a) MIPOSS, (b) MIPOSS-2Br, (c) MIPOSS-Br and (d) MIPOSS-NHBu in DCM solution (10^{-4} M).

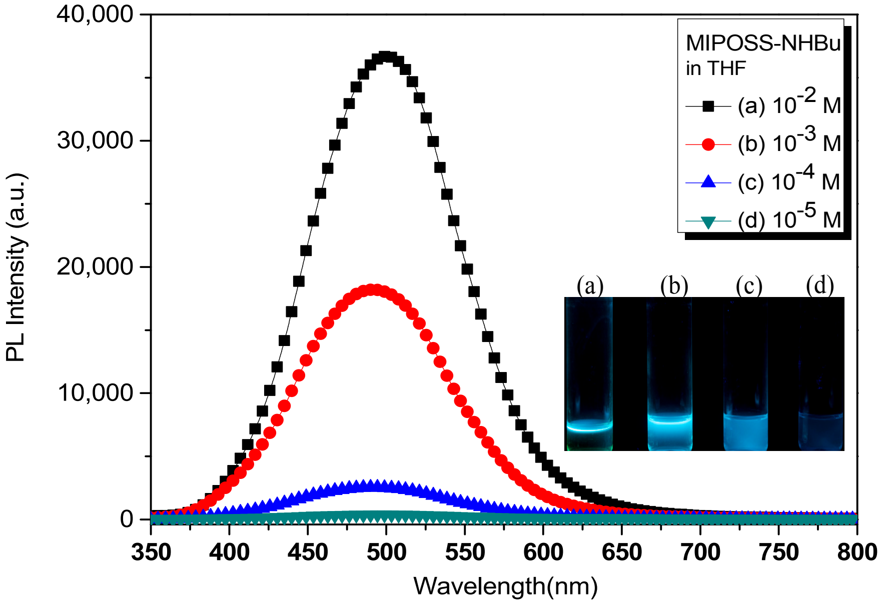

Figure S9. Fluorescence spectra of the monomer MIPOSS-NHBu in THF solution at concentrations from 10^{-5} to 10^{-2} M (excitation: 330 nm).

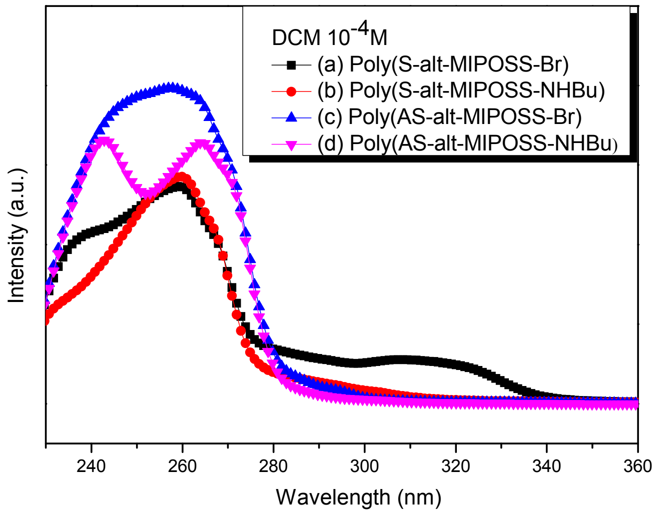

Figure S10. UV-vis spectra of the alternating copolymers (a) poly(S-alt-MIPOSS-Br), (b) poly(S-alt-MIPOSS-NHBu),(c)poly(AS-alt-MIPOSS-Br), and (d) poly(AS-alt-MIPOSS-NHBu) in DCM solution (10-4 M).

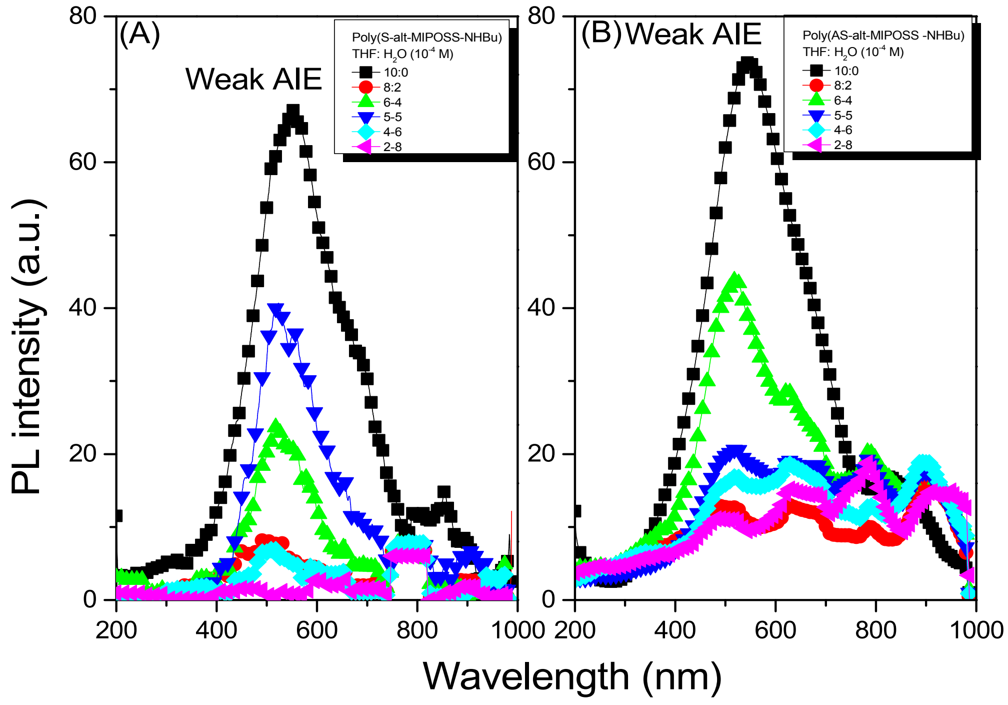

Figure S11. Fluorescence spectra (excitation: 330 nm) of (A) poly(S-*alt*-MIPOSS-NHBu) and (B) poly(AS-*alt*-MIPOSS-NHBu) in THF/H_2O at various ratios, at a concentration of 10^{-4} M.

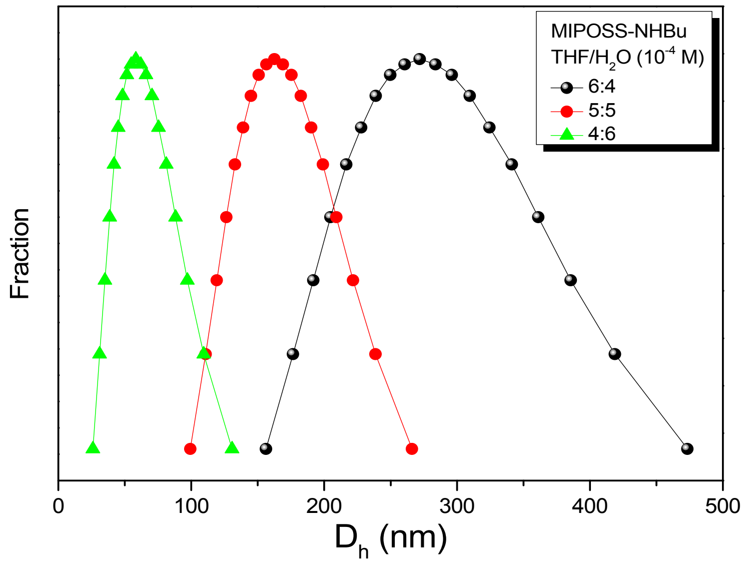

Figure S12. DLS analysis of the monomer MIPOSS-NHBu in THF/H_2O at various ratios, at a concentration of 10^{-4} M.

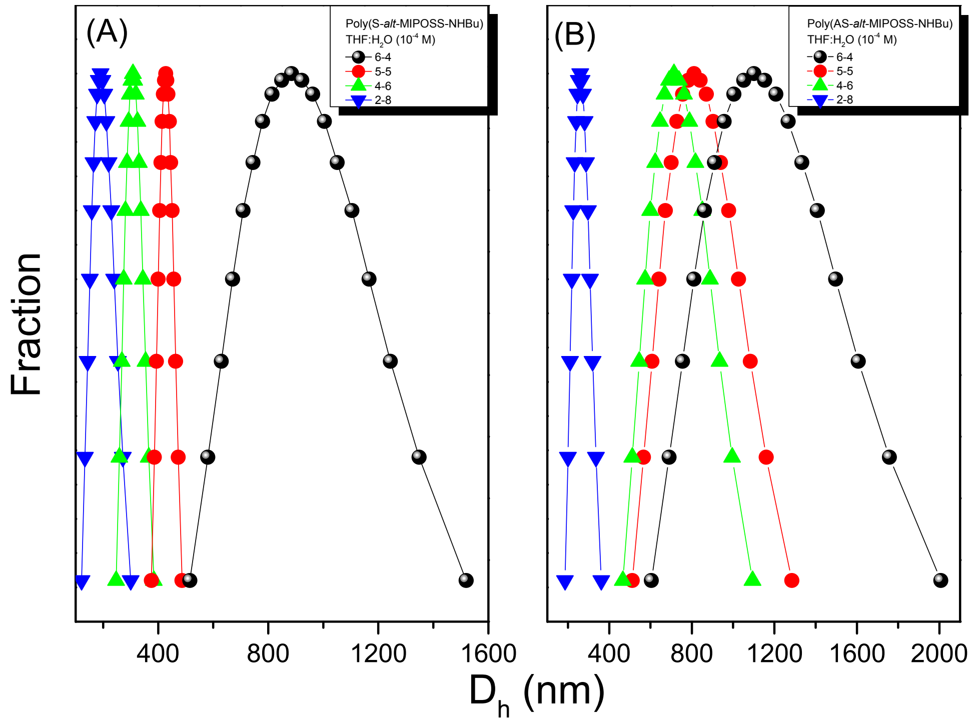

Figure S13. DLS analysis of (A) poly(S-*alt*-MIPOSS-NHBu) and (B) poly(AS-*alt*-MIPOSS-NHBu) in THF/H_2O at various ratios, at a concentration of 10^{-4} M.
